# A Pilot Study Exploring the Feasibility of Virtual Written Exposure Therapy with Underserved Black Perinatal Women

**DOI:** 10.1007/s40615-024-02203-w

**Published:** 2024-10-14

**Authors:** Angela Neal-Barnett, Robert E. Stadulis, Eniolufolake E. Ayoade, Alexis McGhee-Dinvaut

**Affiliations:** Program for Research On Anxiety Disorders Among African Americans, Department of Psychological Sciences, Kent State University, Kent, OH USA

**Keywords:** Black perinatal women, Written exposure therapy, Post-traumatic stress disorder

## Abstract

In the USA, Black pregnant women are at the highest risk for maternal morbidity. They also experience the highest rates of trauma exposure and posttraumatic stress disorder (PTSD). PTSD takes a toll on Black women’s mental and physical health, placing them at risk for maternal morbidity. It increases several mental health symptoms such as suicidality, anxiety, re-living the trauma, and numbness. These mental health conditions adversely affect health behaviors, including those essential for maternal health, such as attending prenatal and postpartum appointments. Furthermore, untreated PTSD is associated with higher blood pressure, increasing Black mothers’ risk of pre-eclampsia. For a variety of reasons including cultural mistrust, stigma, transportation, time constraints, and access to care, PTSD is frequently underassessed and undertreated among Black perinatal women. Written exposure therapy (WET) is a state-of-the-art brief treatment for PTSD. In this study, we explored the initial feasibility of the virtual delivery of WET to reduce PTSD symptoms among Black perinatal women. Results found the virtual delivery of WET to be feasible. Symptom reduction for PTSD in participants was 50–100% during follow-up, suggesting potential effectiveness of the intervention. Implications for virtual delivery of WET in reducing risk for Black maternal morbidity are discussed.

## Introduction

In the USA, Black perinatal women are three to four times more likely to die from pregnancy-related issues [1–3]. Black women are also twice as likely to experience severe maternal morbidity. Whereas a great deal of research has been conducted concerning the role physical health and healthcare play in perinatal maternal morbidity, until recently little attention has been paid to the role of mental health.

Prior to pregnancy, many perinatal Black women have experienced traumatic events placing them at elevated risk for post-traumatic stress disorder (PTSD). Traumatic events include exposure to death, actual or threatened serious injury, or actual or threatened sexual violence [4]. Exposure may occur directly, by witnessing the trauma, by learning that someone was exposed to it, or by indirect exposure usually during professional duties.

Recent research has found that in certain forms, racism meets criteria for trauma [5, 6]. Compared to their White counterparts, Black women have a fourfold risk of entering pregnancy with PTSD and remaining affected [7–10]. Suicidality, re-experiencing the trauma, avoidance, and anxiety are commonly experienced symptoms [11]. Despite the deleterious consequences, screening for trauma and PTSD is not a routine part of maternal healthcare. Among Black perinatal women, PTSD symptoms go unreported and unrecognized. An oft-repeated phrase heard in the authors’ research is as follows: “This has a name, I just thought it was the way things were supposed to be.” [12–14].

Perceiving PTSD symptoms as normal is just one barrier to treatment. Within many Black communities, access to perinatal mental health services is rare and complicated by cultural mistrust. When services are available, transportation, frequent moves/evictions, stigma, and fear of losing custody of one’s children often prevent mothers from seeking help. Time is also a consideration, as many perinatal mothers have limited availability [15]. Current forms of PTSD treatment average between 8 and 15 sessions [15], approximately one trimester. Finally, research has shown that perinatal Black women are more likely to benefit from mental health services delivered by other Black women [16]. Unfortunately, the number of Black mental health specialists working in mental health, let alone perinatal mental health, is disparagingly small. Given the minimum number of Black women delivering services, their availability may be limited. A potential to overcome this barrier to perinatal mental health services might be virtual therapy.

Given the elevated risk for PTSD and the cultural barriers, an innovative approach to PTSD treatment in Black perinatal populations is needed. The ideal intervention would be brief, address cultural mistrust and stigma, increase the likelihood of success by using Black female interventionists, and reduce housing and transportation concerns. During the COVID-19 pandemic, there was a greater reliance on digital communications. CARES ACT funding was used by many local and state officials to increase Wi-Fi access to underserved areas. Thus, in many cities, the infrastructure is in place for the virtual delivery of PTSD interventions.

Written exposure therapy (WET) is a brief therapy that consists of five sessions that focus on 30 min of expressive writing about a traumatic event with the aim of reducing emotional avoidance symptoms [17, 18]. Recent studies have shown that it is as effective as cognitive processing therapy [15]. Originally intended to be delivered in-person, nascent research suggests that virtual delivery WET has similar treatment outcomes to in-person delivery [18]. A key component in virtual delivery is the therapist’s presence [19]. Research has shown that delivering WET through an app without human interaction resulted in a 57% non-completion rate [19]. Based on current research, the use of WET appears to have many advantages for Black perinatal mothers. It can be delivered virtually, eliminating transportation issues, and is briefer than in-person delivery, reducing the burden on mothers’ time. The written component of WET may also help decrease stigma around receiving therapy.

In this study, we examine the feasibility of virtual delivery WET for a community sample of Black perinatal mothers. In doing so, we seek to understand if virtual WET could be implemented with this population, identify if there are any technical difficulties that need to be addressed, determine if participants would complete the five sessions, and determine if WET had the potential to be effective.

## Methods

### Participants

**Fig. 1:**
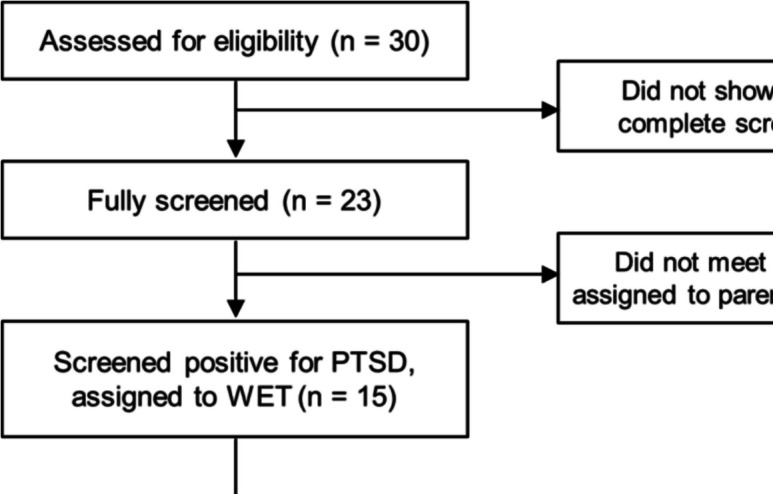
Participant flow chart from enrollment to analysis

This study involved a convenience sample of pregnant and postpartum Black women, residing in Northeast Ohio, specifically in Cleveland, Akron, and Canton, who volunteered to participate in the project. These women were at risk due to low incomes, inadequate housing, insufficient prenatal care, limited access to education and mental health services, and experiences of racism within healthcare. Initially, 30 women expressed interest in participating in the study. Ultimately, 12 women, self-identified as Black, met PTSD criteria and participated in the study. Figure 1 illustrates the evolvement of the final sample. The participants who met criteria to receive WET were nine women who were in the postpartum phase, with the remaining three women pregnant at the time of the intervention. The sample’s demographics are presented in Table 1.

**Table 1:** Sample demographics WET participants (*n* = 12)

Variable	*n*/range	%
Age range in years	25–40	
Number of children born	0–5	
Maternal status		
Pregnant	3	25.0
Postpartum	9	75.0
Previous therapy		
Yes	11	91.7
No	1	8.3
Suicide risk		
Present	3	25.0
Absent	9	75.0
Medicaid recipients	12	100.0

### Measures

#### The Life Events Checklist for DSM-5 – Standard [20]

The Life Events Checklist consists of 16 items known to potentially result in PTSD and one item assessing any other extraordinary stressful event. Level of exposure to the event is rated on a 6-point scale and type of exposure (direct or witnessed) is assessed.

#### Clinician-Administered PTSD Scale for *DSM-5* [21]

Widely used, the Clinician-Administered PTSD Scale (CAPS-5) is a structured interview that can be used to make current (past month) diagnosis of PTSD, make lifetime diagnosis of PTSD, and assess PTSD symptoms over the past week. In addition to assessing the *DSM-5* PTSD symptoms, the interview targets the onset and duration of symptoms, subjective distress, impact of symptoms on social and occupational functioning, improvement in symptoms since a previous CAPS administration, overall response validity, overall PTSD severity, and specifications for the dissociative subtype.

#### UConn Racial/Ethnic Stress and Trauma Survey [22]

The UConn Racial/Ethnic Stress and Trauma Survey (UNRESTS) is a clinician-administered interview that evaluates the impact of experiences of racism throughout a participant’s life. The UNRESTS allows one to determine a PTSD diagnosis because of racism. The semi-structured interview also provides a quantitative assessment of racial/ethnic identity.

#### Subjective Units of Distress

A self-reported scale assessing distress from 0 (no distress) to 100 (greatly distressed). Subjective Units of Distress (SUDS) ratings were used during the WET sessions.

### Procedure

Four Black female doctoral graduate students in clinical psychology and one Black female doctoral student in counselor education served as screeners and interventionists. Interventionists were trained by the first author and a sixth-year graduate student in clinical psychology. Training included both didactic and role-play scenarios. Clinical supervision was provided by the Ph.D. licensed psychologist. Participants were screened by phone or a HIPAA compliant video platform for PTSD using the CAPS-5, UNRESTS, and Life-Events-Scale. For the screenings and WET sessions, participants were instructed to be in a private room without anyone else present. All complied. In addition, interventionists checked to make sure all participants had access to Wi-Fi and either a mobile device, tablet, or computer with a camera, paper, and either a pen or pencil. All had the materials and devices needed to participate in the study. If a participant did not have access to the technology and materials needed, they would have been provided by our team. Upon completion of the screening, participants received a $40.00 e-gift card.

After the screening, the 15 mothers who met DSM-5 criteria for PTSD were invited to participate in WET (see Fig. 1). Thirteen mothers, 12 Black and one Eastern European refugee with a biracial infant, agreed to and participated in WET. The clinical decision was made to offer one mother who met criteria for PTSD the option of either doing WET or grief recovery [23]. Another mother who was about to enter her 8th month of pregnancy and in consultation with her interventionist declined the intervention as she wanted to focus on the pregnancy. The Eastern European refugee’s WET data are not included in the results reported herein. Participants not meeting DSM-5 PTSD criteria (*n* = 8) were referred to our Sisters Offering Support (SOS) sister circle program for stress and anxiety [24, 25] and all agreed to participate. The SOS [24, 25] option was delivered by Black female community health workers-doulas.

WET sessions took place via a HIPAA compliant video platform with each session typically lasting between 45 and 60 min. The WET intervention was delivered as set forth in the manual [17]. Sessions began with a manualized introduction and instructions. The interventionist confirmed that the information was understood by the participant. Next, a SUDS rating was taken, and the participant completed 30 min of writing about the trauma. After the 30 min was completed, the interventionist conducted a check-in about the writing lasting no more than 10 min. On rare occasions, a grounding technique was used. At the end of the check-in, the interventionist wrapped up the session using the manualized script, and the participant took a picture of their written narrative and sent it to a secure email. From screening to the completion of WET, participants were engaged for an average of 7 weeks. Follow-up assessments took place 2–6 months after completion of the original intervention. Upon completing two WET sessions, participants received a $25.00 e-gift card. An additional $25.00 e-gift card was received at the end of the fourth session. Upon completion of WET, participants received a 3-month supply of diapers; upon completion of the follow-up interview, mothers received a $50.00 e-gift card.

### Analysis Plan

As this was a feasibility study, descriptive and inferential statistical assessment of the data were performed. In addition, CAPS-5 pre- and post-total scores were assessed via dependent *t*-test to see if the WET intervention was effective. Pre- and post-session distress scores were compared via a 2 (post) by 5 (WET session) repeated measures ANOVA to determine if each session resulted in a change in distress and if distress was reduced over the five sessions. Follow-up data was only assessed descriptively due to the further reduced sample size.

## Results

As Table 2 indicates, CAPS-5 scores evidenced decline in frequency and severity from pre-WET to post-WET sessions. Score declines were assessed via dependent *t*-test to examine the predictive effect of the WET intervention. Participants’ mean frequency change evidenced a significant decline in PTSD: *t* (11) = 5.90, *p* < 0.001,* d* = 0.79. Similarly, reported severity also decreased significantly, *t* (11) = 5.00, *p* < 0.001, *d* = 0.61.

**Table 2:** CAPS pre- vs post-descriptive statistics

Measure	*n*	*M*	S	SE
CAPS # items pre	8	10.1*	3.09	1.09
CAPS # items post	8	4.4*	4.87	1.72
CAPS severity pre	8	29.0*	12.90	4.57
CAPS severity post	8	13.5*	12.46	4.40

*Pre-post difference, *p* < 0.001

**Fig. 2:**
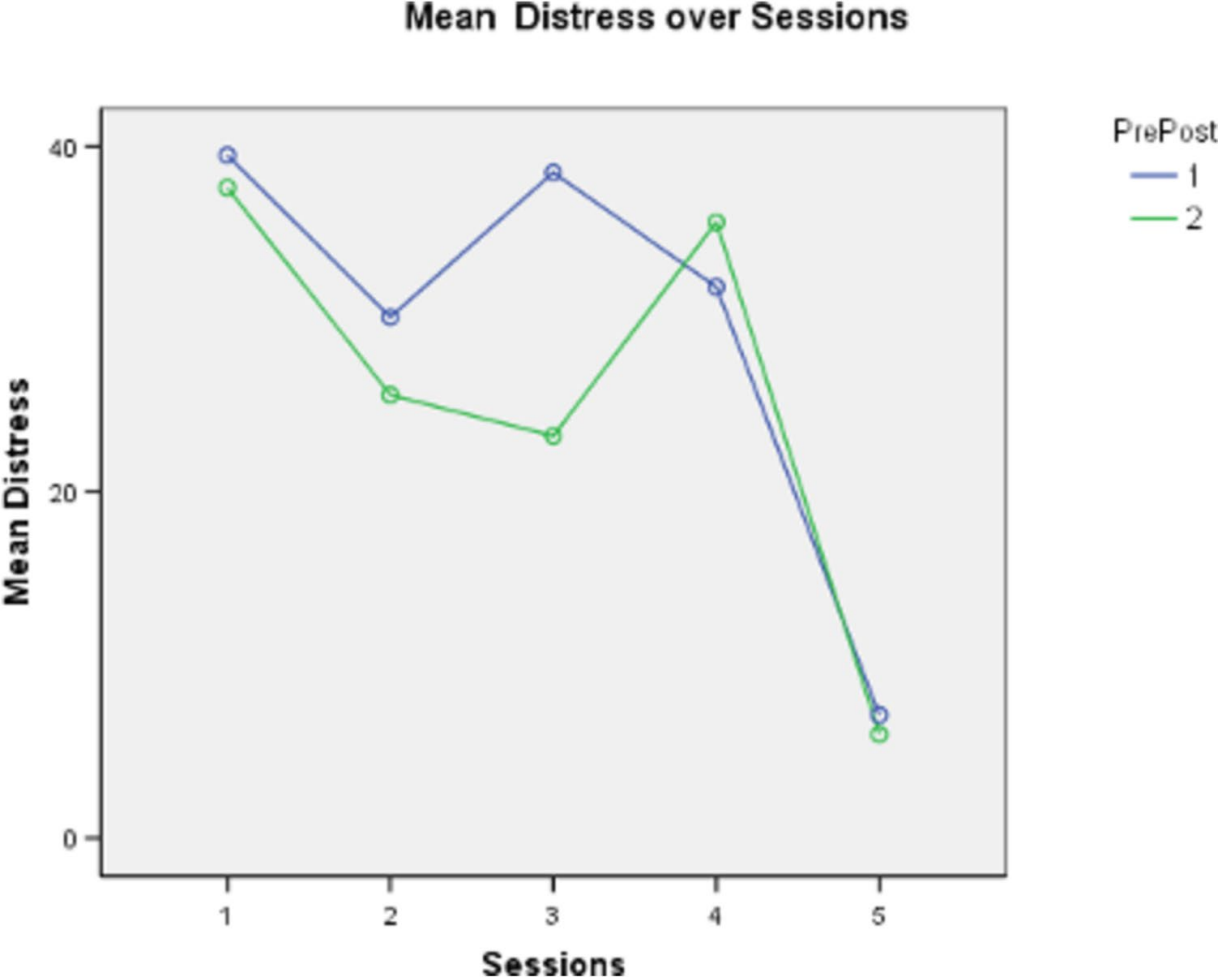
Showing decreased distress over the five sessions

SUDS ratings indicating distress decreased from the first session to the last of the five WET sessions (Fig. 2) with some variability noted in sessions # 3 and #4. At the beginning of the first session, the mean distress of the participants was 40.1 (*S* = 29.3). At the end of the fifth and final WET session, mean distress = 12.8 (*S* = 21.8). A repeated measures ANOVA (Greenhouse–Geisser) 2 (pre vs post) by 5 (sessions) yielded a main effect due to sessions, *F* (4 & 78) = 3.46, *p* = 0.015, *d* = 0.24.

Fifty percent (6 of 12) who completed WET sessions were re-administered the CAPS-5 at least 2 months after the last WET session. Current severity of the WET identified trauma was assessed and compared to severity at the intervention’s conclusion. Results indicated mean severity after completion of WET = 14.8 (*S* = 11.9) whereas the follow-up mean indicated less severity *(M* = 13.0, *S* = 15.4). Thus, the average severity of the identified traumas did not seem to intensify and even slightly decreased further.

We also assessed whether any new trauma had occurred since WET had concluded. The number of identified traumas ranged from 0 to 6, with an average of 4.67 (*S* = 5.5) compared to a mean of 4.92 (*S* = 4.3) on the previous post-session assessment. This represented a small decrease of identified traumas for post-assessment to follow-up.

## Discussion

Results from this study demonstrated the feasibility of the virtual delivery of WET to a community sample of perinatal Black women with PTSD. Twelve Black perinatal mothers completed the five-session intervention with Black female therapists. It is important to note that two additional participants who meet criteria for PTSD but did not undertake WET did so in consultation with our team. The first was a mother who was also experiencing unresolved grief. Grief and trauma are often intertwined [23]. The clinical decision was made by the supervising psychologist to offer this mother a choice between grief recovery or WET. The participant chose grief recovery. The second participant was in the late stages of her third trimester. In discussion with her interventionist about WET, this participant made the decision to focus on her pregnancy and delivery. The East European participant’s WET data is not included in the reported data.

The 12 WET Black participants felt that the intervention was helpful and effective. Each indicated that their symptoms had improved and this was confirmed by the follow-up administration of the CAPS-5. The six follow-up participants each shared their desire to be involved in any other intervention program we had available. The flexibility associated with virtual sessions appeared to play a key role in feasibility. Unlike in-person sessions that must be scheduled within an organization’s hours of operation, virtual sessions could be scheduled outside that timeframe. Furthermore, rescheduling a session was easier. Interventionists could reschedule for a different time the same day or later in the week. The ability to do so seemed to strengthen the interventionist–participant relationship. Rescheduling did not occur regularly, but when it did, mothers felt the interventionist was attentive to their needs. From a feasibility perspective, internet access was not a problem. All participants had access within their homes and internet connections were robust.

In addition to being feasible, virtual delivery of WET had the potential of being effective in reducing PTSD. Among the 12 participants, PTSD symptoms were reduced by 50–100%. Several factors may contribute to these findings. At the core of WET is written narratives; storytelling and narratives are an integral part of Black American culture. Thus, WET may seem both natural and culturally appropriate to our sample. This may also be attributed in part to being seen by same race-same-sex interventionists. This match engendered trust as team members were perceived as women who inherently understood the mothers’ experiences and as “women of their word.” For example, due to a home eviction, a participant had to discontinue. After making sure she was safe and connecting her with housing resources, the interventionist indicated that she was always welcome to return. Nine months later, she did so, telling the interventionist, “I knew you’d still be here for me.”

### Limitations

Given this study’s reliance on a true community sample, it is essential to acknowledge that the sample size is small. However, the strong effect sizes (0.61 and 0.79) for PTSD symptom changes suggest promise for similar future findings in a larger control study. Our study was limited to participants insured by Medicaid, representing only one segment of a Black perinatal community sample. As socio-economic status is a non-protective factor in Black maternal morbidity, the inclusion of privately insured women would add to our knowledge base regarding WET’s feasibility and effectiveness.

Our follow-up rate (50%) was somewhat low. The transient nature of our sample may have contributed. As subsequent studies are considered, tools and techniques to increase follow-up participation should be fully integrated into the research design. Although virtual delivery of WET provides notable advantages in terms of accessibility and convenience, it is worth noting that it may not fully capture the richness of in-person interactions. The absence of real-life engagement might potentially moderate the depth of therapeutic rapport and the nuances of non-verbal communication that shape intervention outcomes. The current study was an open trial, there was no control group and participants were aware of the intervention. These considerations underscore the importance of randomized control studies (RCT) that can explore these factors, ultimately contributing to a more nuanced and well-rounded understanding of the feasibility and potential effectiveness of virtual WET within the Black perinatal community.

## Conclusions

Access to effective perinatal mental health plays a critical role in reducing Black maternal morbidity. In this study, the demonstrated feasibility and promising finding related to effectiveness of the virtually delivered WET intervention raises possibilities for further studies on a larger scale. As more insight is gained about the process variables that may make virtual delivery feasible, research can be expanded to include other perinatal mood and anxiety disorders such as postpartum depression and antenatal anxiety.
